# Broadening horizons: the multifaceted role of ferroptosis in breast cancer

**DOI:** 10.3389/fimmu.2024.1455741

**Published:** 2024-11-27

**Authors:** Anqi Ge, Wang Xiang, Yan Li, Da Zhao, Junpeng Chen, Pawan Daga, Charles C. Dai, Kailin Yang, Yexing Yan, Moujia Hao, Bolin Zhang, Wei Xiao

**Affiliations:** ^1^ The First Hospital of Hunan University of Chinese Medicine, Changsha, Hunan, China; ^2^ Department of Rheumatology, The First People’s Hospital Changde City, Changde, Hunan, China; ^3^ People's Hospital of Ningxiang City, Ningxiang, China; ^4^ Psychosomatic Laboratory, Department of Psychiatry, Daqing Hospital of Traditional Chinese Medicine, Daqing, China; ^5^ Department of Physiology, School of Medicine, University of Louisville, Louisville, KY, United States; ^6^ Tong Jiecheng Studio, Hunan University of Science and Technology, Xiangtan, China; ^7^ Department of Internal Medicine, University of Louisville, Louisville, KY, United States; ^8^ Department of Oral and Maxillofacial Surgery, University of Maryland School of Dentistry, Baltimore, MD, United States; ^9^ Fischell Department of Bioengineering, A. James Clark School of Engineering, University of Maryland, College Park, MD, United States; ^10^ Hunan University of Chinese Medicine, Changsha, Hunan, China; ^11^ Hainan Normal University, Haikou, China

**Keywords:** breast cancer, iron metabolism, ferroptosis, reactive oxygen species, inflammation

## Abstract

Breast cancer poses a serious threat to women’s health globally. Current radiotherapy and chemotherapy regimens can induce drug-resistance effects in cancer tissues, such as anti-apoptosis, anti-pyroptosis, and anti-necroptosis, leading to poor clinical outcomes in the treatment of breast cancer. Ferroptosis is a novel programmed cell death modality characterized by iron overload, excessive generation of reactive oxygen species, and membrane lipid peroxidation. The occurrence of ferroptosis results from the imbalance between intracellular peroxidation mechanisms (executive system) and antioxidant mechanisms (defensive system), specifically involving iron metabolism pathways, amino acid metabolism pathways, and lipid metabolism pathways. In recent years, it has been found that ferroptosis is associated with the progression of various diseases, including tumors, hypertension, diabetes, and Alzheimer’s disease. Studies have confirmed that triggering ferroptosis in breast cancer cells can significantly inhibit cancer cell proliferation and invasion, and improve cancer cell sensitivity to radiotherapy and chemotherapy, making induction of ferroptosis a potential strategy for the treatment of breast cancer. This paper reviews the development of the concept of ferroptosis, the mechanisms of ferroptosis (including signaling pathways such as GSH-GPX4, FSP1-CoQ1, DHODH-CoQ10, and GCH1-BH4) in breast cancer disease, the latest research progress, and summarizes the research on ferroptosis in breast cancer disease within the framework of metabolism, reactive oxygen biology, and iron biology. The key regulatory factors and mechanisms of ferroptosis in breast cancer disease, as well as important concepts and significant open questions in the field of ferroptosis and related natural compounds, are introduced. It is hoped that future research will make further breakthroughs in the regulatory mechanisms of ferroptosis and the use of ferroptosis in treating breast cancer cells. Meanwhile, natural compounds may also become a new direction for potential drug development targeting ferroptosis in breast cancer treatment. This provides a theoretical basis and opens up a new pathway for research and the development of drugs for the prevention and treatment of breast cancer.

## Highlights

- Ferroptosis represents a unique form of iron-dependent cell death, typically characterized by the accumulation of iron and lipid peroxidation.
- This process is implicated in the development and progression of various subtypes of breast cancer, particularly triple-negative breast cancer.
- In experimental tumor models, ferroptosis has shown a dual role in breast cancer, as it can both promote tumor growth and serve as a potential therapeutic target.
- Ferroptosis-inducing therapies are emerging as a promising new strategy for the treatment of breast cancer.

## 1 Introduction

Breast cancer is one of the most common malignant tumors in women and a leading cause of cancer-related deaths among women (1, 2). In 2020, there were 2,261,419 new cases of breast cancer globally, with 684,996 deaths reported (1, 2). In China, there were 416,371 new cases and 117,174 deaths due to breast cancer (1, 2). According to the International Agency for Research on Cancer (IARC), with the aging population and lifestyle changes, the incidence of breast cancer is projected to increase by more than one-third by 2040, reaching over 3 million cases annually, and the mortality rate is expected to increase by over half, with over 1 million deaths annually (1, 2). Various factors influence the development of breast cancer in a patient, with family history being one of the strongest determinants in breast cancer development (3). Breast cancer is a highly heterogeneous tumor, exhibiting diversity in histological morphology and genomic differences, categorized based on histological morphology into non-invasive carcinoma, early invasive carcinoma, and invasive carcinoma, and further subtypes such as lobular carcinoma, ductal carcinoma, intraductal papillary carcinoma, papillary carcinoma, and medullary carcinoma; based on genomic differences, subtypes like Lumina A, Lumina B, HER2, triple-negative breast cancer (TNBC) are identified (4).

Ferroptosis, a distinct form of cell death, is triggered by the iron-dependent accumulation of reactive oxygen species (ROS), leading to mitochondrial lipid peroxidation (5–7). It is characterized by mitochondrial shrinkage, increased membrane density, and nicotinamide adenine dinucleotide (NADH) depletion, without consuming adenosine triphosphate (ATP) (8, 9). In recent years, ferroptosis has emerged as a promising therapeutic target, especially for tumors resistant to conventional treatments (10). For instance, DMOCPTL (a derivative of natural product parthenolide) binds to glutathione peroxidase 4 (GPX4), upregulating early growth response 1 (EGR1) and inducing mitochondrial-mediated apoptosis, effectively inhibiting breast tumor growth (11). In Gefitinib-resistant cells, silencing GPX4 enhances ferroptosis by increasing ROS and malondialdehyde (MDA) levels (12). Additionally, glycyrrhetinic acid induces ferroptosis by promoting ROS and reducing GPX4 and GSH activity in breast cancer cells (13). Genes like miR-5069 and glutathione-specific gamma-glutamylcyclotransferase 1 (CHAC1) also regulate ferroptosis, inhibiting tumor growth through the modulation of ROS and iron-dependent pathways (14, 15). Ferroptosis is particularly significant in the luminal androgen receptor (LAR) subtype of triple-negative breast cancer, where GPX4 inhibitors combined with immunotherapy hold potential as a novel treatment strategy (16).

This review summarizes the current understanding of the ferroptosis regulation, including the prerequisites for ferroptotic responses and the defense systems against iron dysregulation. It delves into the mechanistic basis of breast cancer cell death and presents a conceptual framework for exploiting cell death as a vulnerable target for breast cancer treatment. Finally, several key issues and challenges in future breast cancer cell death research are highlighted, offering more profound insights into breast cancer treatment and prevention.

## 2 Ferroptosis

In 2003, Dolma et al. discovered a novel compound called erastin that can selectively kill engineered tumor cells through a non-apoptotic form of cell death (17). Subsequently, Yang et al. found that this form of cell death could be inhibited by iron chelators, while the compound RSL3 also induced a similar characteristic of cell death (18). In 2012, Dixon et al. discovered that erastin could inhibit the influx of cysteine into cells, leading to a reduction in cellular synthesis of glutathione precursor, inactivation of GPX4, and consequent cell death, thus officially naming this new form of cell death as ferroptosis (19). In 2018, the Cell Death Committee proposed to define ferroptosis as a regulated cell death (RCD) subtype. Cellular ferroptosis is a cell death process regulated by multiple genes and is involved in the regulation of many physiological processes in organisms. Research has found that ferroptosis plays a certain role in ischemic stroke, degenerative diseases (e.g. Alzheimer’s disease and Parkinson’s disease), brain hemorrhage, and other diseases. The regulation process of ferroptosis is mediated by various pathways, such as iron metabolism, System Xc-, lipid metabolism, and mitochondrial VDAC, usually accompanied by the accumulation of excess iron ions and ROS, and the binding of iron ions with hydrogen peroxide, affecting the organism’s microenvironment and leading to cell death. The main manifestations of ferroptosis include decreased mitochondrial volume, increased membrane density, elevated membrane potential, and decreased or disappearing mitochondrial cristae, with no cytoplasmic membrane rupture, and the cell nucleus size remains unchanged (20).

### 2.1 Mechanism of ferroptosis

**Figure 1:**
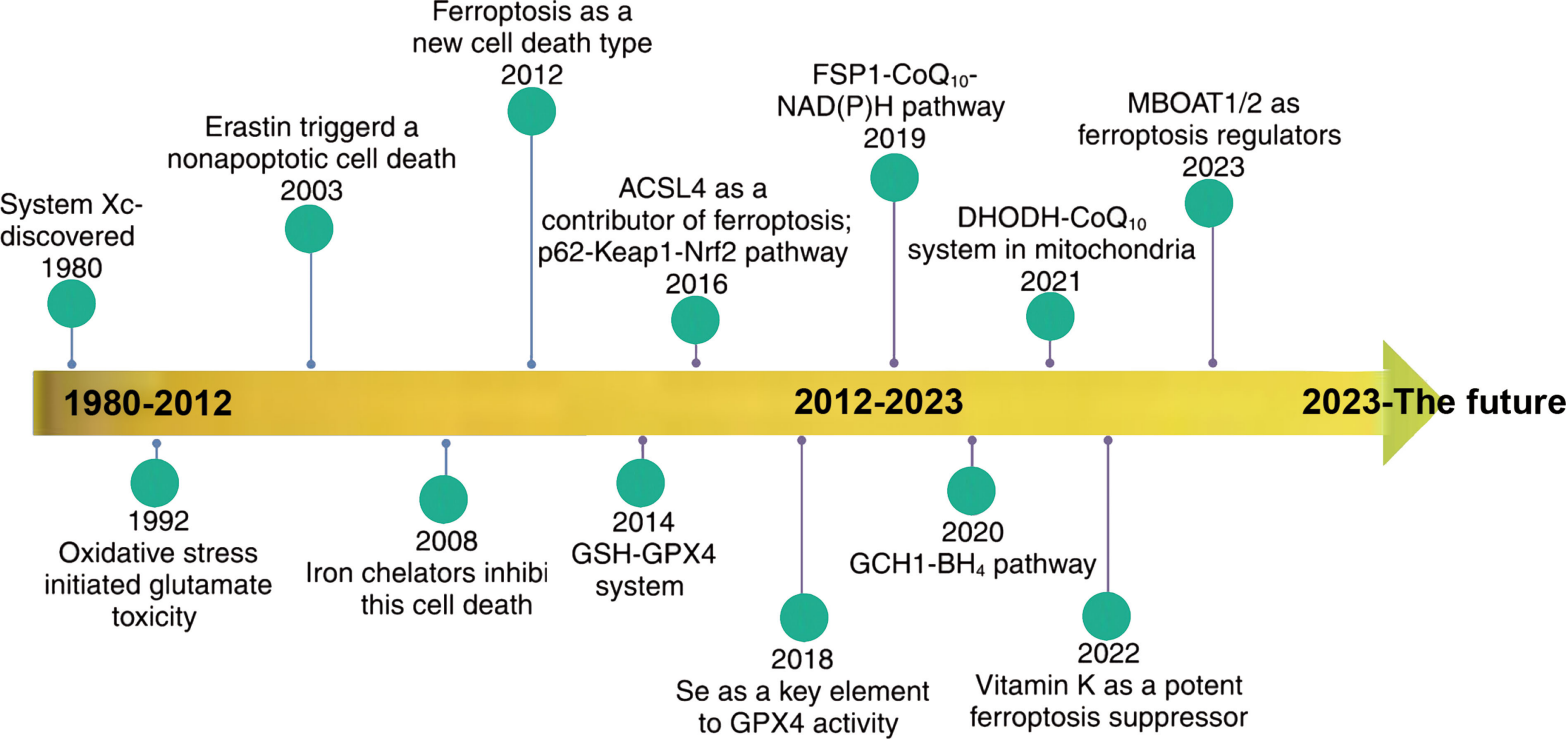
Timeline of ferroptosis research. {figures adapted from Sun et al., 2023 (21), licensed under CC BY 4.0}.

Ferroptosis, as a novel form of cell death distinct from apoptosis and necrosis, is closely associated with various diseases such as ischemic stroke, atherosclerosis, and kidney diseases, and has been widely regarded as a target for tumor therapy. The timeline of ferroptosis research is shown in **Figure 1**.

#### 2.1.1 Iron metabolism

**Figure 2:**
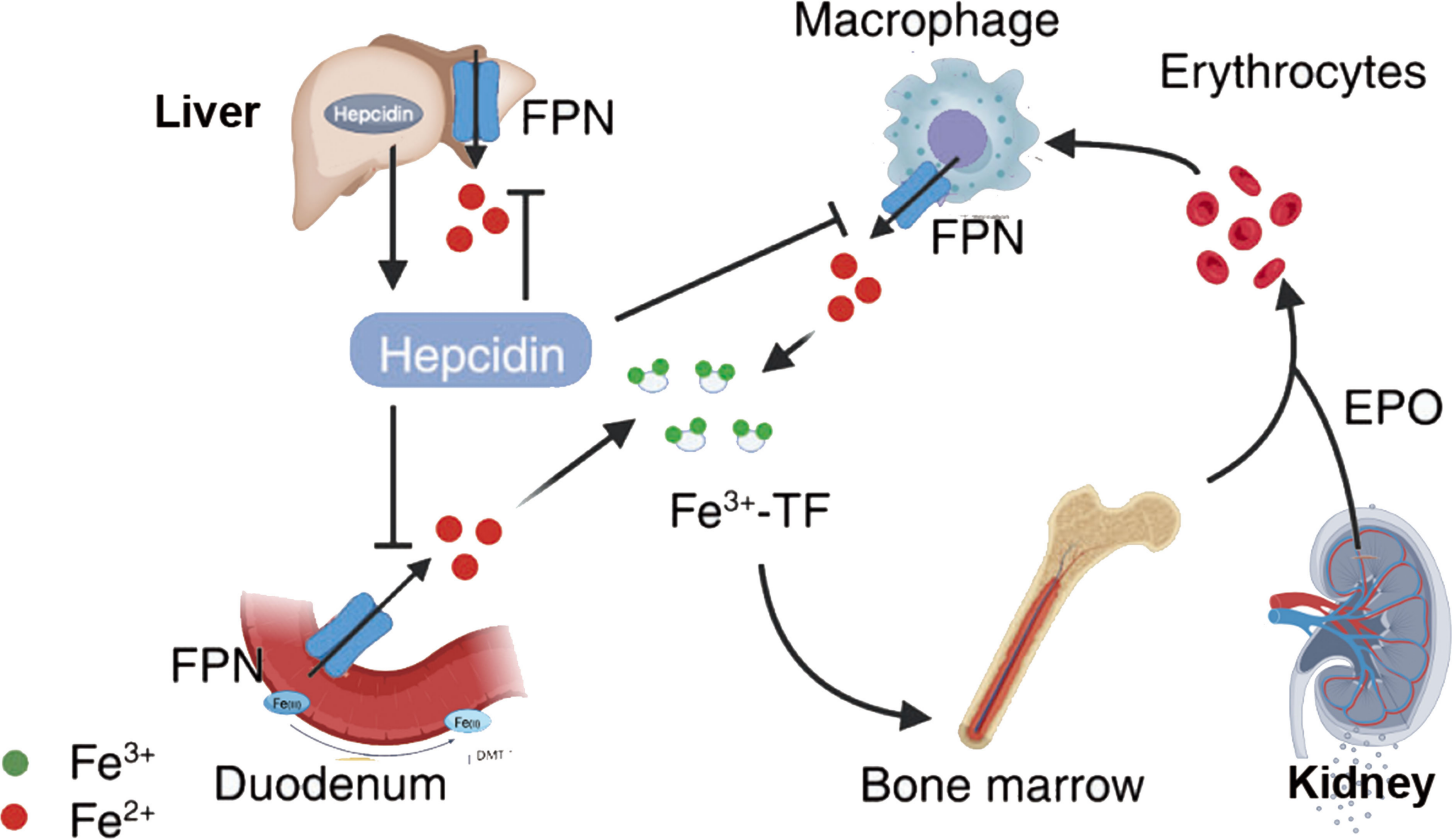
Iron metabolism in ferroptosis. {figures adapted from Sun et al., 2023 (21), licensed under CC BY 4.0}.

Iron is one of the most critical metal elements in animals, and its close relationship with animal metabolism is due to its nature as a transition metal, facilitating electron gain and loss in redox reactions. The dynamic changes in iron redox reactions can enhance the sensitivity of cells to ferroptosis (**Figure 2**). Insufficient iron content in the body may lead to iron deficiency, damaging the synthesis of iron-containing proteins in the body; excess iron may result in iron overload, catalyzing peroxidation reactions leading to the accumulation of excessive ROS within cells, thereby promoting DNA and mtDNA damage. Transferrin (TF) is a serum glycoprotein and a key protein associated with ferroptosis, generally exerting its function by binding with the transferrin receptor (TfR) (22). TF transports iron ions into cells to support cellular growth and plays a role in cell differentiation. TfR has high affinity for iron ions. The first type of transferrin receptor (TfR1) is selectively expressed on the cell membrane in the form of homologous dimers (23), and the second type of transferrin receptor (TfR2) could be divided into full-length (α) and shorter (β) forms, with α type selectively expressed in hepatic cells and erythrocyte precursor cells, while β type primarily exhibits low-level expression in the cytoplasm of various cells. TfR1 and TfR2-α receptors effectively transport iron ions bound to TF into cells (24). The pathways of TF involvement in cellular iron metabolism are as follows: ferrous ions are oxidized by ceruloplasmin to ferric ions in cells, Fe^3+^ binds with apotransferrin (ATF) to form TF-Fe^3+^, then iron-loaded transferrin (holo-TF, TF-Fe^3+^ • hTF) combines with TfR1 to form a complex transported into the cellular endosome. Within the endosome, Fe^3+^ is reduced to Fe^2+^ by Six transmembrane epithelial antigens of prostate 3 (STEAP3). Under acidic conditions, iron ions dissociate from TF and enter cells, subsequently stored in unstable iron pools and ferritin. The entire process is mediated by ferrous ion membrane transport protein 1 (DMT1) or solute carrier family 39 (metal ion transporter), member 8/14 (ZIP8/14) of the zinc-iron protein family (25). Studies have shown that overexpression of TfR can elevate cellular iron ion levels, while TfR silencing can protect cells from ferroptosis (22). Some proteins can modulate the expression of TF and TfR to affect cellular iron ion concentrations and inhibit ferroptosis. For instance, heat shock protein family B (small) member 1 (HSPB1) can suppress TfR1 expression, reduce cellular iron ion concentration, and significantly inhibit ferroptosis when overexpressed (26). Ferroportin (FPN) is an iron-regulating transporter protein encoded by the SLC40A1 gene, primarily involved in iron ion metabolism. FPN regulates iron release in the body, preventing iron ions from inducing ROS formation. In iron-sensitive cells undergoing ferroptosis, FPN expression is reduced. Inhibiting the expression of the main transcription factor for iron metabolism, iron-responsive element binding protein 2 (IREB2), can significantly increase the expression of ferritin light chain (FTL) and ferritin heavy chain 1 (FTH1), thereby inhibiting erastin-induced ferroptosis (27). The transcription of genes regulating iron ion levels mentioned above can influence the abundance of iron ions in the body and the sensitivity of cells to ferroptosis (**Figure 2**).

#### 2.1.2 Lipid metabolism

**Figure 3:**
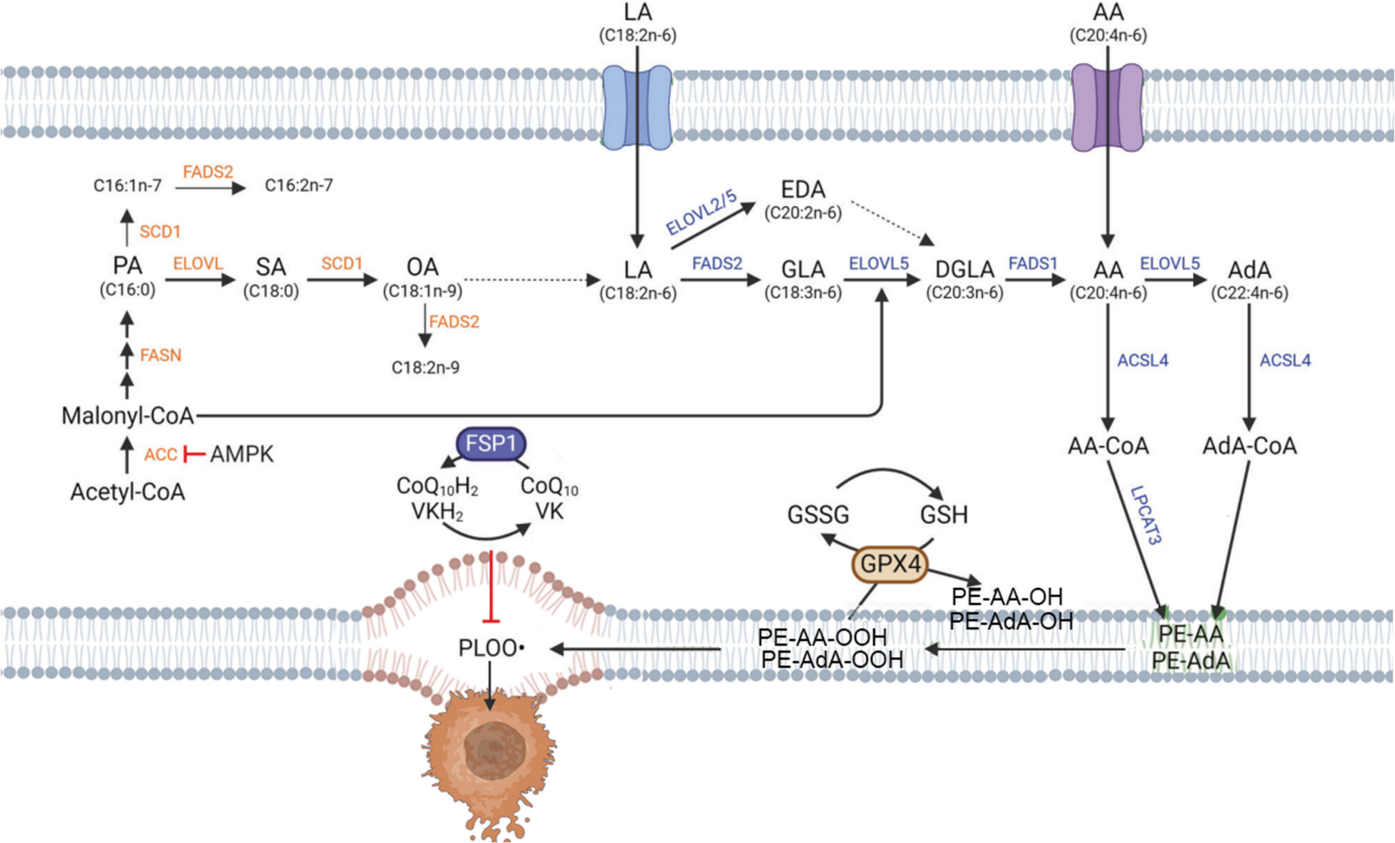
Lipid metabolism in ferroptosis. {figures adapted from Kim et al., 2023 (28), licensed under CC BY 4.0}.

Lipid metabolism affects the sensitivity of cells to ferroptosis and the extent of cellular ferroptosis (**Figure 3**). Polyunsaturated fatty acids (PUFAs) contain diene propyl hydrogen atoms sensitive to lipid peroxidation reactions, which are a crucial step in the process of ferroptosis (29). Monounsaturated fatty acids (MUFAs) play different roles in ferroptosis than PUFAs. PUFAs, such as arachidonic acid, are critical substrates for lipid peroxidation in ferroptosis, whereas MUFAs structurally resist lipid peroxidation and can inhibit ferroptosis (30, 31). The phospholipid content also affects the occurrence of ferroptosis. Members of the long-chain acyl-CoA synthetase family 4 (ACSL4) and lysophosphatidylcholine acyltransferase 3 (LPCAT3) can bind PUFAs to cell membrane phospholipids, promoting cellular ferroptosis. Research shows that ACSL4 is an important pro-ferroptosis gene, and lack of ACSL4 disrupts lipid metabolic balance in cells. Knocking out the genes involved in phospholipid synthesis, like LPCAT3, decreases the material for lipid metabolism, increasing cellular resistance to ferroptosis (32, 33). Stearoyl-CoA desaturase (SCD) and acyl-CoA synthetase long-chain family member 3 (ACSL3) can mediate the generation or activation of MUFAs, replacing PUFAs and inhibiting ferroptosis (34). Lipoxygenases (LOXs) are iron-containing enzyme effector proteins that mediate lipid peroxidation in ferroptosis. LOXs are more likely to bind free unsaturated fatty acids than phospholipid-containing PUFAs. LOXs can oxidize phosphatidyl ethanolamine (PE) acylated by LPCAT3 into toxic lipid hydroperoxides, and knocking out LOXs can alleviate erastin-induced ferroptosis damage (35). Vitamin families and flavonoid preparations have been shown to inhibit LOXs activity and act as ferroptosis inhibitors. The above research indicates that ACSL4, LPCAT3, and LOXs can lead to the accumulation of lipid peroxides, promoting the occurrence of ferroptosis (**Figure 3**).

#### 2.1.3 Mitochondrial voltage-dependent channel

Mitochondria are one of the most important organelles in eukaryotic cells. They consist of two membranes and serve as cells’ primary site for aerobic respiration. The voltage-dependent anion-selective channel (VDAC) is a transmembrane channel that transports ions and metabolites, playing a crucial regulatory role in ferroptosis. The mammalian VDAC family consists of three homologous genes (VDAC1, 2, 3). Research has found that the ferroptosis inducer erastin acts on VDAC2 and VDAC3, leading to mitochondrial dysfunction and releasing large amounts of oxidants, ultimately triggering ferroptosis. Studies have shown that the dihydroorotate dehydrogenase (DHODH) in the inner mitochondrial membrane can resist ferroptosis by regulating the generation of CoQH2 in the mitochondrial inner membrane (36), and other studies have found that mitochondria can regulate ferroptosis through the tricarboxylic acid (TCA) cycle (37). These studies indicate that mitochondria regulate ferroptosis through VDAC and other regulatory mechanisms, and more research is needed to understand the molecular mechanisms regulating ferroptosis.

#### 2.1.4 Ferritinophagy

**Figure 4:**
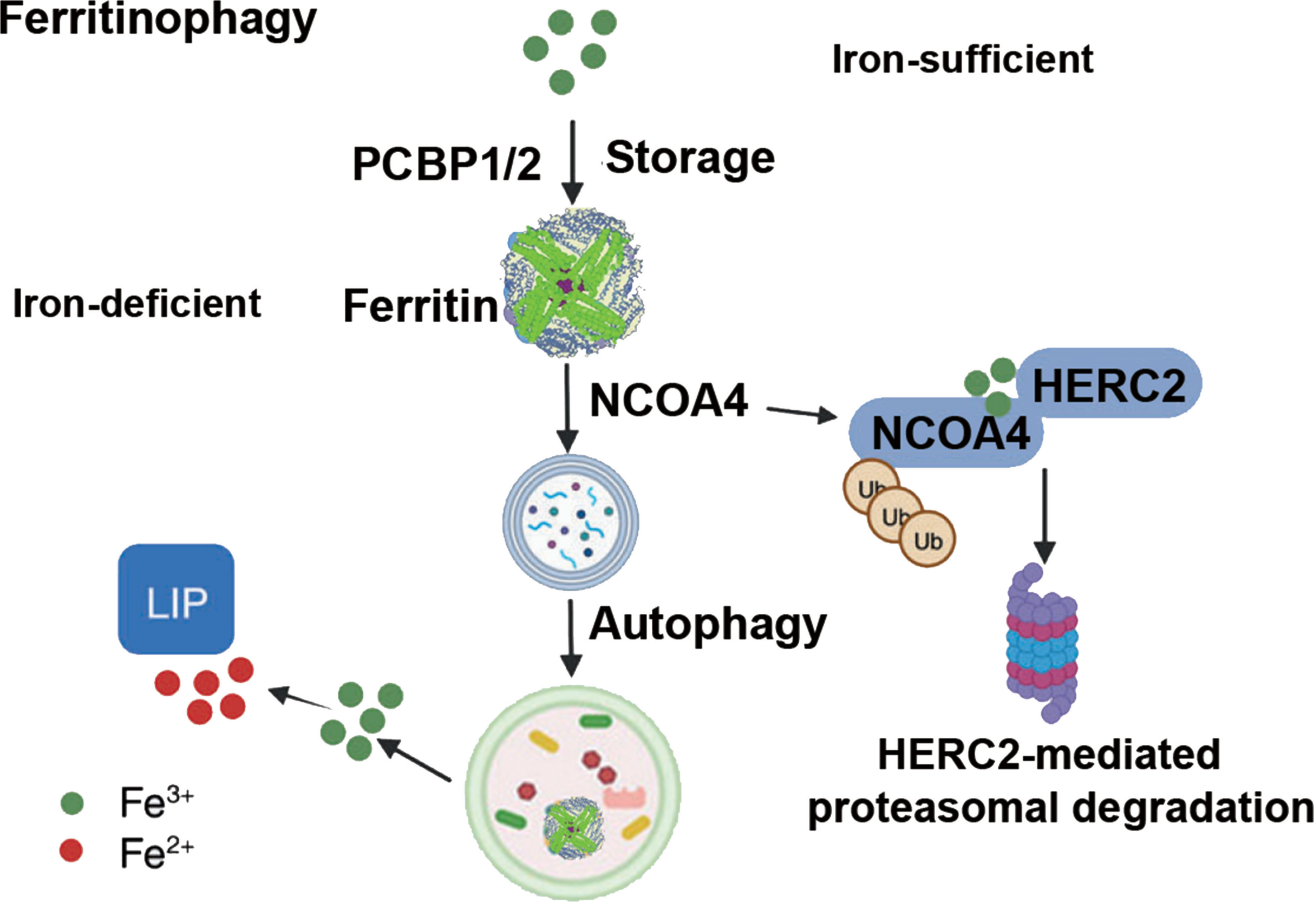
Ferritinophagy in ferroptosis. {figures adapted from Sun et al., 2023 (21), licensed under CC BY 4.0}.

Autophagy, named in 1963, functions through lysosomes to remove damaged organelles and dysfunctional proteins in cells to maintain cellular homeostasis, promote cell metabolism, and self-renewal. Research has shown that various selective autophagic mechanisms can promote the occurrence and development of ferroptosis, such as ferritinophagy, lipophagy, and chaperone-mediated autophagy. Ferritinophagy can regulate the sensitivity of cells to ferroptosis by modulating the abundance of iron ions in cells (**Figure 4**). Specific nuclear receptor coactivator 4 (NCOA4) can mediate ferritin autophagy, and studies have found that inhibiting lysosomal function can block erastin-induced ferroptosis in HT1080 and MEFs cells. NCOA4 binds to microtubule-associated protein 1 (MAP1) light chain 3-phosphatidylethanolamine (LC3-PE) and FTH1, isolating the iron-containing ferritin complex in the autophagosome. Upon autophagosome maturation and fusion with lysosomes, NCOA4 and ferritin are degraded, releasing free iron (38). Adequate free iron participates in biological processes such as hemoglobin synthesis, while excessive free iron promotes cell ferroptosis. Lipophagy degrades lipid droplets in cells via autophagy, inducing lipid release and promoting lipid peroxidation to trigger ferroptosis. Autophagy-related protein 5 (ATG5) and Ras oncogene family member RAB7A are involved in this process. However, tumor protein D52 (TPD52) increases lipid storage to alleviate lipid peroxidation in ferroptosis (39). Therefore, the dynamic balance between lipid synthesis, storage, and degradation in lipophagy regulates ferroptosis. Autophagy can also promote ferroptosis through SLC7A11. Adenosine 5’-monophosphate (AMP)-activated protein kinase (AMPK) is a sensor that maintains autophagy and cellular homeostasis, promoting the phosphorylation of beclin 1 (BECN1) and forming the BECN1-SLC7A11 complex, leading to lipid peroxidation and promoting ferroptosis (40). In summary, more research is needed to further explore the mechanisms through which autophagy regulates ferroptosis.

#### 2.1.5 Other regulatory pathways

Ferroptosis is regulated by the thiol signaling pathway, which helps mitigate oxidative stress through glutathione (GSH) production. Under stress, methionine converts to cysteine, aiding in GSH synthesis and reducing ferroptosis (41). The mevalonate pathway, crucial for synthesizing selenoproteins, also contributes to ferroptosis regulation by supporting GPX4 maturation through isopentenyl pyrophosphate (IPP), a sterol precursor (42). Ferroptosis sensitivity is shaped by processes in organelles, including fatty acid regulation, ROS metabolism, and iron handling (43). In mitochondria, glycolysis and the TCA cycle generate NADH and FADH2, which feed into the electron transport chain (ETC), producing ROS like superoxide anions (O2•−) that contribute to lipid peroxidation. Iron transporters, MFRN1 and MFRN2, facilitate iron metabolism in mitochondria, influencing ROS accumulation (44, 45). Additionally, the OMA1-dependent pathway activates a stress response through ATF4, enhancing GSH metabolism to prevent ferroptosis (46). In the endoplasmic reticulum, lipid synthesis, particularly phosphatidylethanolamine (PE) and phospholipid processing, plays a role in determining cell sensitivity to lipid peroxidation during ferroptosis (47).

### 2.2 Ferroptosis signaling pathway

#### 2.2.1 Glutathione-GPX4 pathway

The most upstream participant in the cascade reaction of ferroptosis signaling is the transmembrane cystine/glutamate antiporter system Xc-, which belongs to the heterodimeric amino acid transporter protein family and acts by replacing extracellular cystine with intracellular glutamate in a 1:1 ratio (48). Cysteine entering the cell is reduced to cysteine by GSH or thioredoxin 1, which is further used in the two-step reactions involving gamma-glutamylcysteine synthetase and glutathione synthetase to biosynthesize the tripeptide GSH (49). Gamma-glutamylcysteine synthetase can be inhibited by L-buthionine sulfoximine (BSO), leading to GSH depletion and cell ferroptosis (50). Current research has shown that inhibiting the input of cysteine required for GSH synthesis leads to the depletion of intracellular GSH levels (51). GSH is a tripeptide antioxidant that serves as a cofactor for the selenium-dependent GPX4 and is a lipophilic hydroperoxide (52). GPX4 can effectively use GSH to reduce phospholipid and cholesterol hydroperoxides to their corresponding alcohols (53). Therefore, consumption of GSH by erastin indirectly inactivates GPX4, accumulating toxic lipid ROS and subsequent lipid peroxidation (51). It is worth noting that early observations dating back to the 1950s and 1970s indicated that deprivation of cysteine inhibited the growth of cell cultures (54), and this type of cell death was inhibited by lipophilic antioxidants and iron chelators (55), directly inhibiting GSH synthesis sufficient to induce ferroptosis in specific cells. For example, the research by Yang and colleagues showed that inhibition of the enzyme glutamate-cysteine ligase (GCL) associated with GSH synthesis with buthionine sulfoximine (BSO) induced cell ferroptosis (56). Additionally, chemical proteomic assays found that the ferroptosis inducer RSL3 covalently binds to the selenocysteine (Sec) at the active site of GPX4, directly inhibiting the peroxidase activity of GPX4 (57). Overexpression of GPX4 in cells can reduce the sensitivity to RSL3-induced ferroptosis, while knockdown of GPX4 promotes cell ferroptosis (56). Other compounds, such as ML162 (56), withaferin A (WA) (58), and the anticancer drug altretamine (59), can also induce ferroptosis by inactivating GPX4.

#### 2.2.2 NADPH-FSP1-CoQ10 pathway

Apoptosis-inducing factor mitochondria-associated 2 (AIFM2), recently identified as an anti-ferrosome gene, has been renamed ferroptosis suppressor protein 1 (FSP1), which can prevent ferroptosis caused by GPX4 deficiency (60). Bersuker and colleagues used CRISPR/Cas9 screening methods to screen for apoptosis in cells treated with RSL3 and a tumor single-guide RNA (sgRNA) library and found that FSP1 can inhibit ferroptosis through a GPX4-independent mechanism (61). FSP1 acts as a nicotinamide-adenine dinucleotide phosphate (NADP)-dependent coenzyme Q oxidoreductase *in vitro* (61). Coenzyme Q10 is a mobile lipophilic electron carrier, an endogenously synthesized lipid-soluble antioxidant, and acts as a lipophilic radical-trapping antioxidant (RTA) in the membrane (62). Research by Doll et al. showed that acylation of the *N*-terminus of the FSP1 protein with myristic acid helps localize FSP1 to the membrane (63). FSP1 acts as an oxidoreductase on the membrane to reduce coenzyme Q (CoQ) to ubiquinol (CoQH2), which then acts as a lipophilic radical-trapping antioxidant to reduce the toxicity of lipid peroxide radicals (61). FSP1 is a parallel ferroptosis regulatory mechanism to GPX4, inhibiting ferroptosis by modulating non-mitochondrial coenzyme Q. The combination pharmacological inhibition of FSP1 and GPX4 may be an effective strategy to sensitize cancer cells, especially those insensitive to GPX4 inhibitors alone, to ferroptosis-inducing chemotherapeutic agents (64).

#### 2.2.3 GCH1-BH4 pathway

Guanosine triphosphate (GTP) cyclohydrolase 1 (GCH1) is the rate-limiting enzyme in the conversion of GTP to 7,8-dihydroneopterin triphosphate (NH2TP), followed by phosphoribosyl transferase (PTS) and sepiapterin reductase (SPR) enzymes converting NH2TP to tetrahydrobiopterin (BH4) (65). BH4 is an oxidation-reduction active cofactor involved in producing nitric oxide, neurotransmitters, and aromatic amino acids (66). Previous research on BH4 focused on its antioxidant properties *in vitro* (67). Recently, Kraft et al. used a genome-wide activation library for CRISPR/dCas9 overexpression screening and identified the protective gene GCH1 and its metabolite BH4 as ferroptosis inhibitors. Their study showed that cells expressing GCH1 could inhibit ferroptosis by inducing lipid remodeling through BH4 synthesis (68). Although GCH1 overexpression conferred strong protection against RSL3 and IKE-induced ferroptosis and GPX4-induced ferroptosis, it did not protect cells from apoptosis inducers and is only slightly effective against necroptosis. This finding suggested that GCH1 selectively counteracts ferroptosis cell death (68). Soula et al. also demonstrated that deficiency of GCH1 or SPR leads to decreased levels of BH4 in Jurkat cells, making them sensitive to RSL3, but GCH1 deficiency does not make cells sensitive to erastin or other compounds inducing ROS. This indicates that GCH1 and its metabolite BH4 are effective endogenous ferroptosis inhibitors, serving as an independent and effective pathway for ferroptosis separate from the GPX4/GSH pathway.

#### 2.2.4 E-cadherin-NF2-Hippo-YAP signaling pathway

The Hippo-Yes-Associated Protein (Hippo-YAP) signaling pathway involves various biological functions, including regulating cell proliferation and organ size (69). Researchers have investigated the role of this pathway in ferroptosis and observed that high-density cells are more resistant to ferroptosis induced by cysteine deficiency and GPX4 inhibition (70). Mechanistically, the cell density effect on epithelial cell ferroptosis is mediated by E-cadherin-mediated cell-cell contact, activating the Hippo signaling pathway through NF2 (also known as Merlin) tumor suppressor protein, inhibiting YAP factor nuclear translocation and transcriptional co-regulation (71). The role of this pathway in ferroptosis begins with an exciting study: under cysteine starvation and GPX4 inhibitor induction, densely growing cells are more resistant to ferroptosis (70). This result is similar to some earlier significant findings, such as cells grown at extremely high density or cultured in spheroids (which facilitate cell-cell contact) enhancing the survival of GPX4-knockout cells. Mechanistically, the regulation of epithelial cell ferroptosis by cell density is mediated via E-cadherin-mediated cell-cell contact, facilitating cell interactions through the activation of the intracellular Hippo signaling pathway by the epithelial cell calcium-binding protein NF2 (also known as Merlin), thus inhibiting the nuclear translocation and transcriptional activity of the oncogene YAP. Since YAP targets multiple ferroptosis regulatory factors, including ACSL4 and the transferrin receptor TfR1, the occurrence of ferroptosis ultimately depends on the activity of the Hippo pathway; inhibiting the Hippo pathway and activating YAP can both promote ferroptosis (69, 72). Consistent with this, studies have found that the homolog of YAP, TAZ, promotes ferroptosis in renal cancer cells (which mainly express TAZ rather than YAP) by regulating cell density. The role of the E-cadherin-NF2-Hippo-YAP pathway in regulating ferroptosis is of significant importance (69).

#### 2.2.5 AMPK signaling pathway

Current research indicates that energy and metabolic stressors can lead to energy loss, resulting in the systemic cascade failure necessary to maintain internal balance, such as energy-dependent ion imbalances across cell membranes, ultimately leading to cell death (73). Additionally, subjecting cells to metabolic stress through glucose deprivation can promote the generation of ROS, suggesting that glucose deprivation may enhance ferroptosis (74). Surprisingly, glucose deprivation actually inhibits cell ferroptosis, and this protective effect depends on the activity of the energy-sensing kinase AMPK. Therefore, when glucose is lacking, AMPK is activated, initiating an energy stress protection program to counter ferroptosis, involving impaired biosynthesis of PUFAs, which are essential for lipid peroxidation-induced ferroptosis (75). The activation of the energy stress protection program can protect the kidneys from ischemia-reperfusion injury (IRI). This protective mechanism may be the first line of defense against organ damage caused by energy depletion, as organ damage often accompanies energy depletion (76, 77).

#### 2.2.6 HIF2α-HILPDA signaling pathway

Current research indicates (78) that ferroptosis induced by erastin under 1% oxygen environment is unaffected, suggesting that hypoxia does not inhibit ferroptosis. A recent study (79) demonstrated that hypoxia actually sensitizes cancer cells to ferroptosis, as clear cell carcinoma is highly sensitive to ferroptosis induced by GPX4 inhibitors, driven by the hypoxia-inducible factor 2α (HIF2α) subtype through hypoxia-induced lipid droplet-associated protein (HILPDA) via PUFA lipid remodeling. Clear cell carcinoma histologically exhibits typical clear cytoplasmic staining and is difficult to treat, highlighting the clinical significance of this finding. Therefore, it is speculated that the sensitivity of cell ferroptosis driven by HIF2α-HILPDA is likely an ancient method to eliminate neoplasms with hypoxic characteristics (80).

## 3 Relationship between ferroptosis and breast cancer

Ferroptosis plays a significant role in the growth, proliferation, and death of various tumor cells. It is associated with multiple diseases, and the activation of ferroptosis is observed in various solid tumors (80). Ferroptosis also plays a role in cancer metastasis to bone, a common site for breast cancer spread. As a result, targeting ferroptosis could be a potential immunotherapeutic strategy for managing breast cancer bone metastasis (81). Studies have shown that in tumors such as pancreatic cancer, colorectal cancer, and lung adenocarcinoma, there is an increase in iron utilization and iron overload to maintain a high iron state in cells, which favors tumor growth and metabolism (82). Therefore, understanding the mechanisms of Ferroptosis in tumor cells can play a vital role in diagnosing and treating tumors. Currently, most chemotherapy drugs induce apoptosis in tumor cells, inhibiting their growth and metastasis. However, when tumor cells exhibit resistance mechanisms such as increased drug efflux and decreased apoptosis, they develop resistance to chemotherapy, posing a clinical challenge. As a novel form of non-apoptotic programmed cell death, ferroptosis uniquely regulates tumor initiation and progression. Therefore, activators of Ferroptosis hold great promise in treating tumors (8). The tumor suppressor P53 can promote tumor cell Ferroptosis by inhibiting SLC7A11 or increasing arginase-1 and ornithine decarboxylase 2 expression. Interestingly, P53 can also directly inhibit dipeptidase 4 activity or induce the expression of cyclin-dependent kinase inhibitor 1A to suppress Ferroptosis occurrence (8). We reviewed the research progress on ferroptosis in relation to breast cancer progression, prognostic prediction, and targeted therapy.

### 3.1 Relationship between ferroptosis and breast cancer progression

Ferroptosis, as a downstream common link of many gene regulatory pathways, is closely related to breast cancer progression. In the study by Chen et al. (83), it was found that filamin A interacting protein 1 can interact with SLC7A11, promoting its ubiquitin-mediated proteasomal degradation to induce tamoxifen resistance in breast cancer cells. Lin et al. (84) identified that epithelial-mesenchymal transition-mediated recurrent breast tumors upregulate the discoidin domain receptor tyrosine kinase 2 (DDR2), which maintains the growth advantage of tumor cells while activating YAP/TAZ-mediated ferroptosis sensitivity, explaining the higher ferroptosis sensitivity in recurrent tumor cells. Furthermore, in the triple-negative breast cancer cell line BT549, long-chain acyl-CoA synthetase 1(ACSL1) mediates the synthesis of α-tocopherol succinate (αESA, a representative polyunsaturated fatty acid) and induces ferroptosis in BT549 cells, which cannot be suppressed by GPX4 interference (85). Inhibiting α-tocopherol succinate synthesis provides a novel direction for the treatment of breast cancer resistant to GPX4 inhibitors. Ferroptosis in breast cancer cells is also regulated by many non-coding RNAs. Circular RNA circGFRA1 competitively binds to miR-1228, regulating the expression of the target gene FSP1, and mediating malignant tumor phenotypes (86). Long non-coding RNA PGM5P3-AS1 stabilizes the expression of the target gene microtubule-associated protein light chain C by binding to RNA binding protein NOP58, promoting ferroptosis and inhibiting the malignant progression of triple-negative breast cancer (87). Yadav et al. found that SLC7A11 is a downstream target of miR-5096, and SLC7A11 rescue reduces the ferroptosis mediated by miR-5096 and promotes tumorigenesis (14). There are many other studies on the correlation between non-coding RNAs and ferroptosis in breast cancer, focusing mainly on elucidating molecular relationships and the potential of non-coding RNAs in predicting Ferroptosis occurrence and tumor prognosis. We look forward to synthesising small molecules targeting non-coding RNAs related to ferroptosis to provide more treatment options for breast cancer.

The tumor microenvironment (TME) is a complex entity, and investigating the interactions between tumor cells and other components within the microenvironment is crucial in breast cancer, where ferroptosis also plays a role. Xie et al. (88) discovered a protective role of fat cells on ferroptosis in a co-culture system of fat cells and breast cancer cells. Further research revealed that oleic acid secreted by fat cells can inhibit breast cancer ferroptosis in the presence of acyl-CoA synthetase long-chain 3. Liu et al. found that acute exposure to high 27-hydroxycholesterol led to lipid metabolism disorders in breast cancer cells due to disrupted sterol regulatory element-binding protein signaling, thus inhibiting tumor cell growth (89). On the other hand, breast cancer cells chronically exposed to high 27-hydroxycholesterol exhibited increased tumorigenic and metastatic abilities. These cells upregulated the GPX4 axis activity and reduced System Xc antioxidant system dependency to adapt to the stress of lipid metabolism abnormalities. This study partly explains why obesity and high cholesterol are adverse prognostic factors for tumors. Interestingly, researchers also found a correlation between cell interactions in the TME and Ferroptosis. Interleukin-6 produced by triple-negative breast cancer tumor cells can activate the JAK2/STAT3 axis in tumor-associated macrophages, inducing the secretion of TGF-β1, which in turn activates the TGF-β1 pathway in tumor cells and regulates the expression of hepatocyte leukemia factor (HLF), a novel cancer protein. HLF transcriptionally activates γ-glutamyl transferase 1 to inhibit Ferroptosis, promoting breast cancer growth and chemoresistance. Despite the findings of TME’s impact on tumor progression through the Ferroptosis pathway, most Ferroptosis events are limited to tumor cells (90). Tumor immune escape, as a crucial factor in tumor progression and the theoretical basis for immunotherapy, is characterized by effector T-cell exhaustion induced by tumor cells. It is widely believed to be mediated by programmed cell death, and the relationship between Ferroptosis and immune cell exhaustion remains poorly understood. Although challenging to detect, further research in this area is meaningful for a better understanding of TME.

### 3.2 Mechanism of ferroptosis in the breast cancer

#### 3.2.1 Mechanisms of iron accumulation overload in breast cancer

The tyrosine kinase inhibitor lapatinib is a commonly used chemotherapy drug for HER-2+ breast cancer patients, which can induce iron accumulation and ferroptosis by increasing the expression of transferrin receptor 1 (TFR1) in cancer cells, inhibiting brain metastasis of cancer tissues (91). Similarly, the synergistic anticancer effects of chemotherapy drugs sorafenib and lapatinib can induce ferroptosis in TNBC cells by increasing the transferrin (TF) level and promoting intracellular iron accumulation, demonstrating synergistic anticancer effects (92). Liu et al. discovered that transmembrane protein 189 (TMEM189) can inhibit autophagy in TNBC cancer cells, decrease TFR1 expression and intracellular lipid reactive oxygen species (Lipid-ROS) content, alleviating ferroptosis and promoting breast cancer growth *in vivo*, suggesting that TMEM189 inhibitors may have a better therapeutic effect on TNBC (93). Treatment of TNBC with iron-saturated lactoferrin (Lf) combined with a radiation dose of 4 Gy significantly enhances erastin-induced ferroptosis in TNBC, inhibits cancer cell proliferation and migration capabilities, and enhances radiation sensitivity (94). The patented traditional Chinese medicine, Shuganning injection, could selectively upregulate heme oxygenase 1 (HO-1) expression in TNBC cells, leading to lipid iron pool accumulation and ferroptosis occurrence, inhibiting breast cancer tissue growth both *in vivo* and *in vitro* (95). Artemisinin can promote ferritin autophagy, increase intracellular Fe^2+^ levels, and induce ferroptosis in TNBC cells, enhancing cancer tissue sensitivity to the ferroptosis inducer RSL3 and inhibiting breast cancer development (96). Iron-saturated Lf, Shuganning injection, and artemisinin are potential candidate drugs for treating TNBC.

In recent years, there have been numerous studies reporting on targeted breast cancer treatment using nanotechnology based on the mechanism of iron accumulation-induced ferroptosis. Xu et al. developed a nano-metal-organic framework (MOF-Fe^2+^) with Fe^2+^ as the core, efficiently delivering Fe^2+^ to breast cancer cells, promoting the Fenton reaction and abundant generation of ROS, and inducing ferroptosis (97). This nano framework effectively inhibits breast cancer tissue growth *in vivo*. Zhu et al. (98) constructed a Fe^3+^ cross-linked nanostructure loaded with trans-azobenzene-camptothecin, specifically entering breast cancer cells. Upon infrared light excitation, the loaded Fe^3+^ converts to Fe^2+^, triggering the Fenton reaction and lipid peroxide accumulation, inducing cancer cell ferroptosis. Applying this particle in mice significantly reduces breast cancer volume with good anti-tumor effects. Zhang et al. designed a cascade release nanoparticle (NP) driven by acetylcholinesterase, loaded with doxorubicin, ferrocene (Fc), and TGF-β receptor inhibitor SB431542 (99). Upon entering breast cancer cells, this NP increases intracellular iron accumulation and ROS levels through doxorubicin and ferrocene, activating ferroptosis and successfully preventing cancer tissue metastasis in mice. The clinical efficacy of these NPs needs further verification.

#### 3.2.2 Mechanism of system Xc-/GSH/GPX4 in breast cancer

Inhibiting the activity of the System Xc-/GSH/GPX4 axis is an effective way to prevent breast cancer growth and treat breast cancer (100, 101). Clinical studies have found that the active ingredient dihydrotanshinone I in Salvia miltiorrhiza can inhibit GPX4 expression in breast cancer cells, promote ferroptosis, and inhibit tumor growth in mice without significant side effects (102). The derivative of the natural product chamomile lactone, DMOCPTL, can induce ferroptosis in breast cancer cells by directly binding to GPX4 protein, leading to tumor growth inhibition in mice and extending mouse lifespan significantly without observable toxicity (11). Wen et al. extracted an active compound, glycyrrhetinic acid, from the traditional herb licorice, and found that it could reduce the activity of GSH and GPX4, exacerbate lipid peroxidation, and induce ferroptosis in breast cancer cells (13). Metformin can upregulate the level of miR-324-3p in TNBC cells, targeting to promote ferroptosis and significantly reduce tumor growth *in vivo* (103). Additionally, Jaggupilli et al. discovered a small molecule compound, V9302, that inhibits glutamine uptake and significantly reduces GSH and GPX4 activity in SUM159 and MDA-MB-231 cells, inducing lipid peroxidation and promoting ferroptosis in TNBC cells (104). When used in combination with paclitaxel *in vitro* and *in vivo*, it significantly inhibits breast cancer growth. Dihydrotanshinone I, DMOCPTL, glycyrrhetinic acid, metformin, and V9302 may be effective potential drugs for treating breast cancer. Yao et al. reported that simvastatin (SIM) could weaken 3-hydroxy-3-methylglutaryl-CoA reductase to inhibit the mevalonic acid pathway and GPX4 expression, thereby inducing ferroptosis in TNBC cancer cells (105). They further loaded SIM into magnetic nanoparticles coated with amphiphilic polycations (Fe_3_O_4_@PCBMA) and significantly inhibited breast cancer development *in vivo*. Currently, Fe_3_O_4_ has been approved by the US Food and Drug Administration (FDA) for clinical use, and the Fe_3_O_4_@PCBMA-SIM nano-system is supposed to have great potential for clinical application.

Li et al. found that tumor-associated macrophages could upregulate HLF expression in TNBC cells by secreting transforming growth factor beta 1, increasing breast cancer tissue volume and decreasing sensitivity to chemotherapy drug cisplatin (90). Mechanistically, HLF transcriptionally activates γ-glutamyl transpeptidase, which catalyzes GSH shearing in the extracellular space and increases GSH content in cancer cells, thereby inhibiting ferroptosis, enhancing the proliferative and invasive ability of TNBC cells, and resistance of cancer tissue to cisplatin. Zou et al. identified fibroblast growth factor receptor 4 (FGFR4) as a necessary gene for HER2-positive breast cancer to acquire resistance; inhibiting FGFR4 in mice can reduce GSH synthesis and efflux efficiency of Fe^2+^ through the β-catenin/TCF4-SLC7A11/FPN1 axis, leading to excessive ROS production, LIP iron accumulation, and ferroptosis, enhancing the sensitivity of drug-resistant HER2-positive breast cancer to chemotherapy drugs (106). HLF and FGFR4 may be potential drug development targets for preventing and treating breast cancer. Novel breast cancer prevention and treatment materials targeting GSH and ferroptosis have been reported in recent years. A sono-dynamic sensitizer containing the platinum (II)-indole complex can reduce GSH levels in TNBC cell line 4T1, promote ROS generation and ferroptosis, and enhance cancer tissue’s sensitivity to ultrasound radiation therapy (107). Zhou et al. used cinnamaldehyde to synthesize a dimer that can deplete GSH (108). Combined with sorafenib, it significantly enhances the ferroptosis of 4T1 cancer cells and successfully eradicates breast cancer in mice by promoting dendritic cell maturation and CD8+ T cell activation.

The actin-binding protein 1 can directly bind to System Xc- and degrade System Xc- through the ubiquitin-proteasome system, enhancing the sensitivity of MCF7 cells to erastin, and inhibiting breast cancer growth *in vivo* (83). The chemotherapy drug sulfasalazine can induce ferroptosis in breast cancer cells by specifically inhibiting the function of SLC3A1, and the use of this function in treating breast cancer is in Phase II clinical trials (109). Metformin down-regulates SLC7A11 expression by inhibiting UFMylation modification of SLC7A11, promoting ferroptosis in TNBC cancer cells, reducing breast cancer volume *in vivo*, and achieving better results when used in combination with sulfasalazine (110). Liquiritin inhibits the NF-κB signaling pathway in MDA-MB-231 and MCF-7 cells, reduces System Xc- expression, promotes ferroptosis, and alleviates resistance of breast cancer tissue to doxorubicin (111). Trastuzumab can increase the expression of circular RNA BGN in breast cancer cells BT474 and SKBR3, enhance the deubiquitination and expression of SLC7A11, inhibit ferroptosis, induce drug resistance in cancer tissue, an effect that can be reversed by erastin (112).

#### 3.2.3 Lipid metabolism involvement in ferroptosis

The synthesis and peroxidation of polyunsaturated fatty acids-phospholipids (PUFA-PLs) in the cell membrane are prerequisite and key steps for the occurrence of ferroptosis (75). Acyl-CoA synthetase long-chain family member 4 (ACSL4) and lysophosphatidylcholine acyltransferase 3 (LPCAT3) are key enzymes involved in the synthesis of PUFA-PE (113–115). Sha et al. (116) collected biopsy specimens from 199 breast cancer patients who underwent paclitaxel-cisplatin chemotherapy and found through rank tests and Cox proportional regression analysis that the expression of ACSL4 and the combination status of ACSL4/GPX4 can serve as independent predictive factors for pathological complete response, and ACSL4 expression is positively correlated with overall survival in breast cancer patients. Increased expression of ACSL4 in the TNBC cell line MDA-MB-157 enhances the sensitivity of cancer tissue to the ferroptosis inducer RSL3, while deletion of ACSL4 prevents cancer cells from undergoing RSL3-induced ferroptosis and leads to increased tumor volume *in vivo* (117). These findings suggest that ACSL4 may be a potential drug development target for treating TNBC.

In contrast to ACSL4, monoacylglycerol-acyltransferase 3 (ACSL3) or stearoyl-CoA desaturase 1 (SCD1) mediated generation of monounsaturated fatty acids (MUFAs) (e.g., oleic acid) can competitively inhibit PUFA-related ferroptosis by replacing phospholipids in the cell membrane with MUFA-phospholipids. Studies have shown that inactivation of ACSL3 or SCD1 can enhance the sensitivity of breast cancer cells to ferroptosis (118, 119). Luis et al. have reported a significant upregulation of SCD1 and fatty acid-binding protein 4 (FABP4) in human breast cancer specimens, which is associated with poor prognosis in different types of breast cancer (120). Mechanistically, SCD1 catalyzes fatty acid desaturation and synergistically promotes lipid droplet formation with FABP4 in the breast cancer microenvironment, alleviating hypoxia-induced ferroptosis in MDA-MB-231 cells and promoting cancer cell regeneration and recurrence. Conversely, downregulation of SCD1 and FABP4 expression significantly inhibits lipid transport in the cancer tissue microenvironment, induces ferroptosis, and reduces breast cancer recurrence and metastasis. The cannabinoid receptor 1 inhibitor LIMONENE can activate the PI3K and MAPK signaling pathways, reduce MUFA generation mediated by SCD1 and fatty acid desaturase 2, promote ferroptosis in TNBC cells, increase sensitivity of cancer tissue to erastin and RSL3, and limit the growth of breast cancer tissue *in vivo* (121). These findings suggest that LIMONENE may be an effective drug for treating breast cancer, although the specific mechanisms need further investigation and confirmation.

#### 3.2.4 Involvement of the Nrf2/HO-1 signaling pathway in breast cancer mechanisms

Nuclear factor erythroid 2-related factor 2 (Nrf2) is a crucial transcription factor in oxidative stress response that can induce the expression of heme oxygenase-1 (HO-1), inhibit ROS expression, and exert anti-inflammatory and anti-ferroptotic effects. In recent years, it has been discovered that Nrf2 plays a vital role in the progression, treatment, and drug resistance of breast cancer. Jiang et al. found that in breast cancer patients receiving anti-PD-1/PD-L1 therapy, the expression of Tyrosine-Protein Kinase Receptor 3 (TYRO3) in cancer tissues is often positively correlated with poor prognosis (122). Mechanistically, anti-PD-1/PD-L1 treatment can significantly increase the expression of TYRO3 in breast cancer tissues, further elevate intracellular Nrf2 levels, reduce ROS generation, inhibit ferroptosis, create a tumor-promoting microenvironment, and confer resistance to anti-PD-1/PD-L1 therapy. Wu et al. reported decreased glycogen synthase kinase-3β (GSK-3β) expression and a significant upregulation of Nrf2 in breast cancer tissues (123). Overexpression of GSK-3β in TNBC cells can inhibit Nrf2 expression, increase ROS and MDA levels, enhance erastin-induced ferroptosis, and in an *in vivo* breast cancer xenograft model, GSK-3β overexpression enhances the tumor growth-inhibiting effect induced by erastin. Echinomycin A is an active compound extracted from *Ligusticum wallichii*, which can disrupt mitochondrial structure and function in breast cancer cells, enhance ROS-induced ferroptosis in TNBC cells through activation of the Nrf2/HO-1 signaling pathway, and could be a potential lead compound for breast cancer treatment (124). BET inhibitors are commonly used chemotherapy drugs in breast cancer treatment, and NR5A2 and NCOA3 mainly mediate their resistance. Mechanistically, NR5A2 and NCOA3 act synergistically to increase Nrf2 expression and inhibit cancer cell ferroptosis. Using small molecule inhibitors to target NR5A2 or NCOA3 can significantly enhance the anti-cancer effects of BET inhibitors in breast cancer *in vitro* and *in vivo* (125).

#### 3.2.5 Molecular mechanisms of FSP1-CoQH2 involvement in ferroptosis

FSP1 is a glutathione-independent iron suppressor and a novel biomarker for anti-ferroptosis (61, 63). Research has shown that silk fibroin nanoparticles encapsulating rosuvastatin can effectively inhibit the redox enzyme activity of FSP1, thereby slowing the malignant progression of triple-negative breast cancer (126).

#### 3.2.6 Molecular mechanisms of DHODH–CoQH2 involvement in ferroptosis

Dihydroorotate Dehydrogenase (DHODH) is located in the mitochondrial membrane, involved in pyrimidine synthesis, and can reduce CoQ to CoQH2 in the mitochondrial membrane. Activation of DHODH promotes an increase in CoQH2 generation, thereby inhibiting lipid peroxidation in mitochondria and exerting anti-ferroptotic effects (127). The DHODH inhibitor brequinar selectively inhibits tumor growth with low GPX4 expression by inducing ferroptosis. Combined with brequinar and sulfasalazine, it synergistically induces ferroptosis and inhibits tumor growth with low GPX4 expression (36). The DHODH inhibitor siR/IONs@LDH can induce ferroptosis in breast tumors (128).

#### 3.2.7 Epigenetic regulation of ferroptosis in breast cancer

It is known that cysteine is an essential substrate for synthesizing GSH. Therefore, decreased cysteine, GSH, and GPX4 levels can lead to ferroptosis in breast cancer cells, thereby inhibiting tumor growth (129). Generally, GPX4 expression is upregulated in breast cancer, closely related to the increased expression of the two subunits of the cysteine/glutamate antiporter (xCT), SLC7A11 and SLC3A2. Meanwhile, xCT in TNBC can interact with MUC1-C and CD44 variant (CD44v), increasing GSH levels, inhibiting ferroptosis occurrence, and resulting in high proliferation and invasive activity (130). Additionally, various genes/proteins, non-coding RNAs (ncRNAs), and signaling pathways are involved in regulating ferroptosis in breast cancer. For example, miR-5096 enhances intracellular ROS, OH-, lipid ROS, and iron accumulation levels in breast cancer cells by targeting SLC7A11/xCT, reducing GSH and mitochondrial membrane potential, inducing ferroptosis in breast cancer cells, and further inhibiting their proliferation, migration, and invasion abilities (14). miR-324-3p and miR-382-5p induce ferroptosis by targeting GPX4 and SLC7A11’s 3’-UTR, inhibiting breast cancer progression (103, 131). Circular RNA RHOT1 can suppress ferroptosis occurrence through the miR-106a-5p/STAT3 axis, promoting breast cancer progression (132). Silencing glycogen synthase kinase-3β (GSK-3β) with low expression in breast cancer can upregulate nuclear factor erythroid 2-related factor 2 (Nrf2) and GPX4 expression, inhibit tumor cell ferroptosis, and promote progression (133). Stearoyl-CoA desaturase-1 (SCD1) in breast cancer cells can synergistically protect tumor cells with fatty acid binding protein-4 (FABP4) in the tumor microenvironment, shielding them from oxidative stress-induced ferroptosis (134). Overactivation of the PI3K-AKT-mTOR signaling pathway can inhibit ferroptosis occurrence through sterol regulatory element binding protein 1 (SREBP1)/SCD1-mediated lipid synthesis (84). Recurrent breast cancer epithelial-mesenchymal transformation (EMT) regulatory factors TWIST and SNAIL induce tumor cell ferroptosis significantly in a DDR2-dependent manner (135). Additionally, ACSL4 and KLF4 are key molecules in the ferroptosis signaling pathway; they can inhibit ferroptosis in breast cancer cells while maintaining their malignant biological characteristics (116, 136, 137). Tumor suppressor p53’s role in ferroptosis is also interesting: research indicates that its promotion or inhibition of ferroptosis in tumors mainly depends on the surrounding cellular environment. Current studies suggest that p53 primarily promotes ferroptosis in breast cancer, but the specific molecular mechanism requires further investigation (138).

### 3.3 Ferroptosis in breast cancer prognostic prediction

The advancement of bioinformatics has assisted researchers in mining ferroptosis-related genes from tumor databases and constructing prognostic prediction models to improve breast cancer prognosis evaluation systems. Wan et al. selected 11 ferroptosis-related differentially expressed genes from the TCGA database and developed a breast cancer prognostic prediction model (127). Multifactor Cox regression analysis suggested that high-risk scores from this model were independent risk factors for poor prognosis in breast cancer. Similar studies have also been conducted for Luminal-type breast cancer (139) and breast cancer patients undergoing neoadjuvant therapy (102). Additionally, in a clinical study, Sha et al. conducted immunohistochemical testing on biopsy samples from breast cancer patients undergoing paclitaxel and cisplatin neoadjuvant chemotherapy to explore the potential of known ferroptosis-related genes ACSL4 and GPX4 as predictive markers for pathological complete response (140). This study found that high expression of ACSL4, low expression of GPX4, and their combined state were independent prognostic factors for disease-free survival.

In a multicenter imaging multi-omics cohort study, researchers determined different image ITH (IITH) phenotypes and their prognostic value through DCE-MRI imaging genomic features (141). Furthermore, researchers comprehensively validated this heterogeneity assessment method from a mesoscopic to microscopic scale using genomics, transcriptomics, metabolomics, and digital pathology data, revealing the biological behavior of high-IITH tumors. Interestingly, researchers reported that ferroptosis is a susceptibility factor for high-IITH breast cancer and a promising target in the era of precision oncology. HSPB1 inhibits ferroptosis in breast cancer cells and regulates the NF-κB signaling pathway to promote chemoresistance (12). There is a close relationship between ferroptosis and breast cancer prognosis; many differentially expressed genes have been identified as key genes in the ferroptosis pathway, affecting patient clinical outcomes. However, the functions of many genes remain unknown, and further molecular mechanism studies on them can help researchers overcome the limitations of bioinformatics prediction accuracy and identify more therapeutic targets for breast cancer.

### 3.4 Targeting ferroptosis as a therapy for breast cancer

#### 3.4.1 Small molecule compounds inducing ferroptosis

Studies have found that triple-negative breast cancer cells resistant to epidermal growth factor receptor tyrosine kinase inhibitor gefitinib commonly exhibit elevated expression of GPX4 (11). The ferroptosis inducer erastin has shown promising cytotoxic effects on resistant cells, yet its use in humans is limited due to its renal toxicity. Li et al. found that amiodarone inhibits breast cancer cell proliferation by targeting the KAT5/GPX4 axis (142). Additionally, metformin promotes ferroptosis in breast cancer cells by inducing UFMylation modification of SLC7A11, independently of the AMPK signaling pathway, revealing a novel anticancer mechanism of metformin (110). The active component red ginseng polysaccharide in the traditional Chinese medicine ginseng has been found to inhibit GPX4 expression. When used in combination with the ferroptosis inducer erastin, it exhibits better anti-breast cancer growth effects (143). Small molecule compounds (such as elastin and RSL3) currently under development or discovery mainly target the classic System Xc antioxidant system of tumor cell ferroptosis, with a future focus on other non-classical systems such as FSP1-CoQH2 and the regulation of immune cell death in the tumor microenvironment.

#### 3.4.2 Targeted drug delivery for ferroptosis

The main obstacle to applying ferroptosis inducers is their side effects, prompting researchers to explore targeted drug delivery using nanoparticles. Pan et al. constructed a nano-metal-organic framework of Fe-TCPP (TCPP = tetrakis (4-carboxyphenyl) porphyrin) loaded with hypoxia-activable prodrug tirapazamine (TPZ), which was enveloped in breast cancer cell membranes to form a composite of nanoparticles and cancer cell membranes (144). Due to the encapsulation of cancer cell membranes, the composite is not easily cleared by the immune system and can aggregate at the tumor site. Subsequently, the Fe^3+^ within the particles promotes GSH depletion and lipid reactive oxygen species (LipROS) accumulation through the Fenton reaction, inducing ferroptosis. Zhou and co-workers developed a nanocomposite composed of hemoglobin, perfluorocarbon, ferroptosis inducers, and photosensitizer verteporfin, enclosed in red blood cell membrane camouflage, which under ultrasonic stimulation induces ferroptosis in breast cancer cells (145). *In vitro* experiments demonstrated that the nano-complex showed tumor tissue selectivity and better anticancer effects under mild ultrasonic stimulation. Moreover, research on pH-sensitive and photosensitive nanoparticles inducing ferroptosis has been conducted (145, 146). The main strategies for designing such nanoparticles include: (1) using autologous cell membrane encapsulation to overcome immune clearance; (2) inducing ferroptosis through passive accumulation of Fe^3+^ and LipROS and/or disruption of antioxidant systems; (3) exploiting synergistic effects with chemotherapy drugs; (4) designing external activation methods such as ultrasound to enhance targeting.

#### 3.4.3 Increasing sensitivity to treatments through ferroptosis

**Figure 5:**
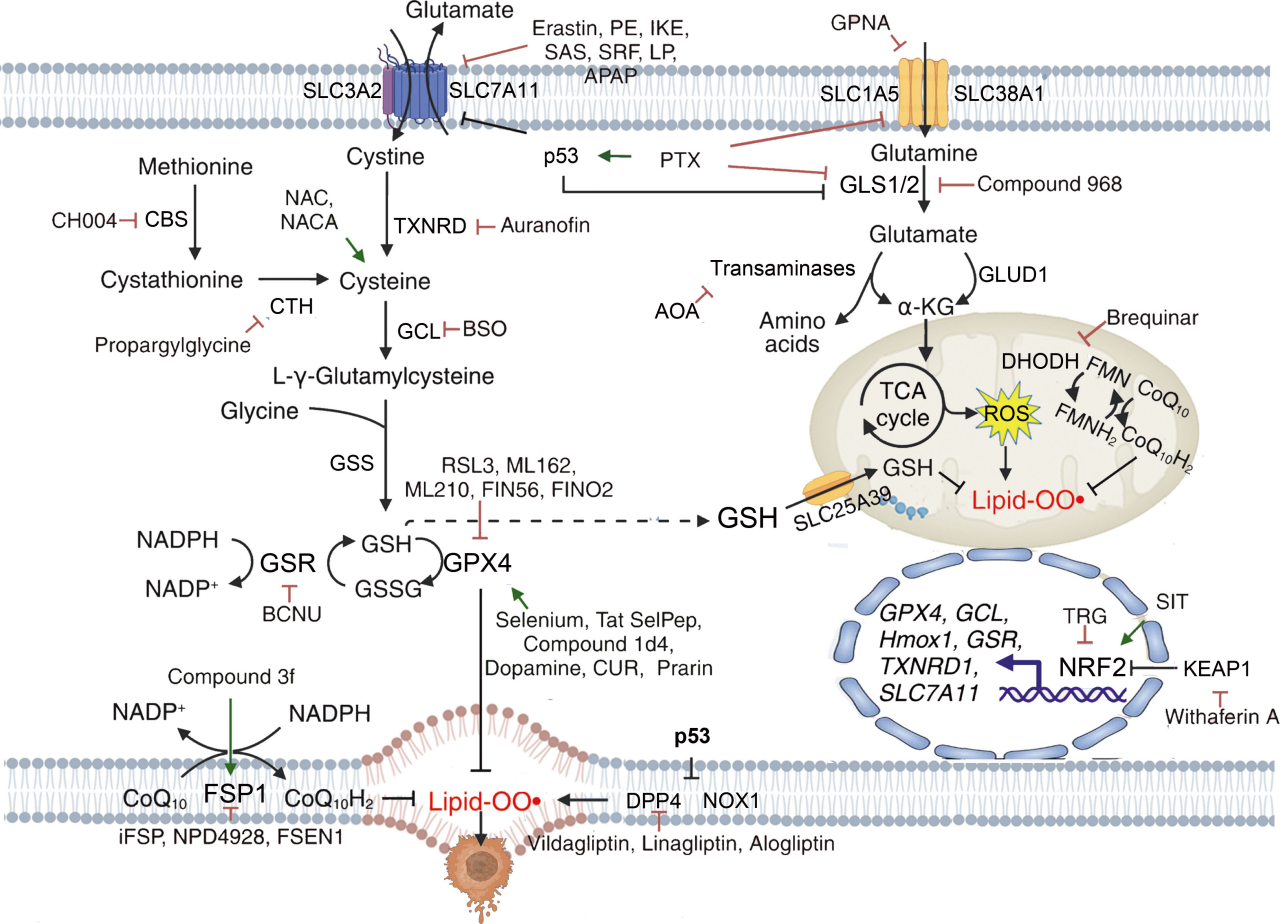
Mechanisms of targeting ferroptosis in reductive-oxidative pathways. {figures adapted from Sun et al., 2023 (21), licensed under CC BY 4.0}.

**Figure 6:**
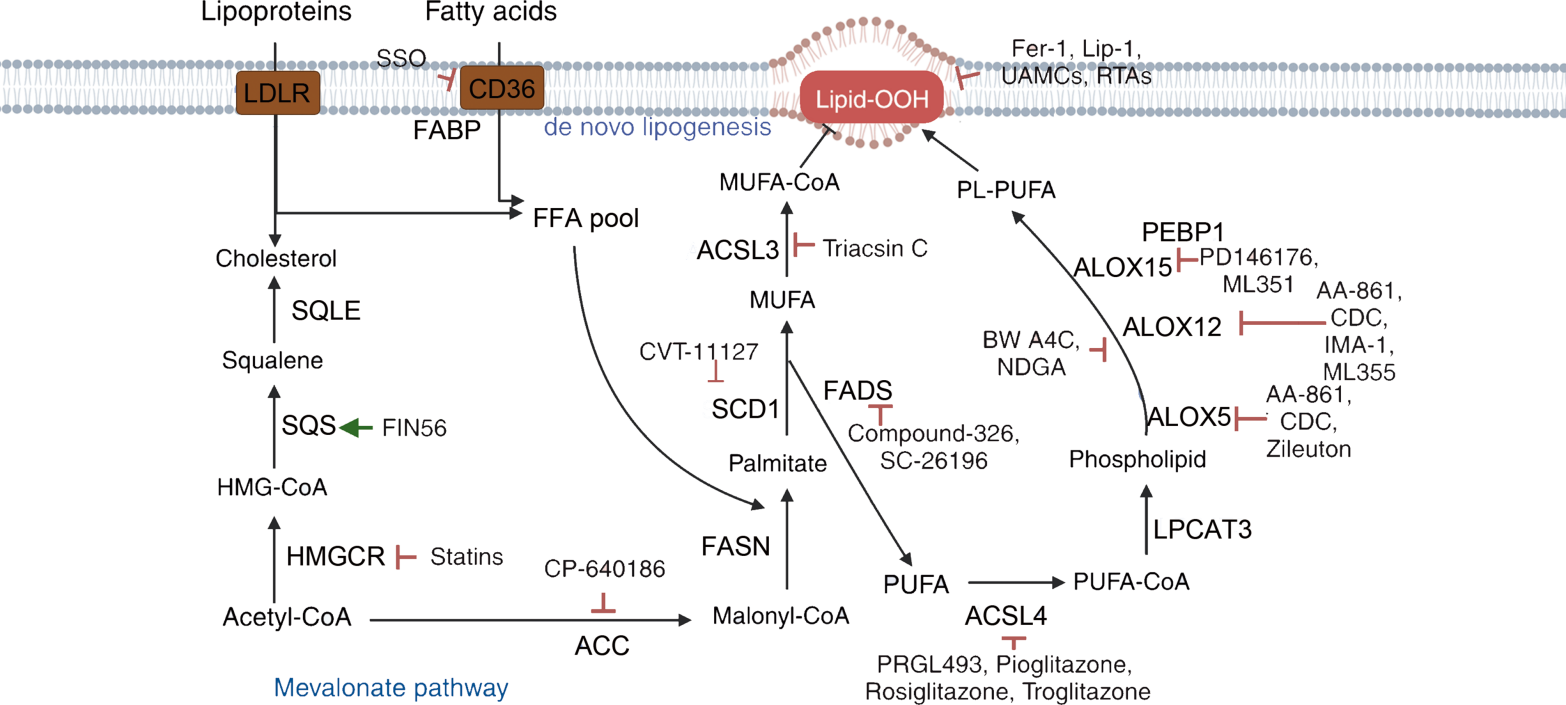
Mechanisms of targeting ferroptosis in lipid metabolism pathways. {figures adapted from Sun et al., 2023 (21), licensed under CC BY 4.0}.

Radiotherapy and drug resistance are the main reasons for the poor efficacy of breast cancer treatment. Zhang et al. found that iron-saturated MDA-MB-231 cells are more sensitive to radiotherapy (147). In addition to ferroptosis, iron-saturated cells downregulate HIF-1α expression, improve the tumor’s hypoxic microenvironment, and promote radiation-induced hypoxic DNA damage. Their research provides a basis for combining radiotherapy with iron-loading treatment. For chemotherapy-resistant cells, the combination of ferroptosis inducers has shown effectiveness, and the future direction is to enhance the safety of clinical applications. Furthermore, Zou and the team found that trastuzumab targets the resistance-related gene FGFR4, inhibiting ferroptosis through the FGFR4-βcatenin/TCF4-SLC7A11 axis and promoting resistance in HER2-positive breast cancer (106). The combination of ferroptosis inducers also showed significant therapeutic effects. The above studies all suggest that the combined application of activating ferroptosis with traditional treatment methods can have a synergistic effect (**Figures 5**, **6**).

## 4 Ferroptosis in TNBC - a highly aggressive form of breast cancer

TNBC is currently the most aggressive molecular subtype of breast cancer, characterized by high heterogeneity and poor prognosis. It accounts for approximately 15% to 20% of all breast cancer cases, and patients with TNBC typically have worse outcomes, with over 50% experiencing recurrence within 3 to 5 years after diagnosis and treatment. This presents significant challenges in terms of comprehensive treatment and prognosis analysis for breast cancer, highlighting the urgent need to develop novel therapeutic drugs and provide more precise interventions for TNBC patients (148, 149).

### 4.1 Inducing ferroptosis in TNBC by selective inhibition of System Xc-

In breast cancer with low estrogen receptor expression, Yu et al. found that sulfasalazine can serve as a System Xc- inhibitor, inducing ferroptosis in breast cancer cells (150). Hasegawa et al. suggested that downregulating the MUC1-C/System Xc- signaling pathway can also inhibit TNBC cell activity, even eliminating TNBC cells, thus controlling tumorigenic cell growth and metastasis (130). Therefore, selectively inhibiting System Xc- in TNBC cells to induce ferroptosis may be a new direction in conquering TNBC.

### 4.2 Targeting GPX4 in TNBC directly

TNBC is a type of tumor rich in iron and lipids, making the induction of ferroptosis crucial for TNBC treatment. Studies have shown that the derivative of PTL, DMOCPTL, can significantly inhibit the proliferation of TNBC cells by ubiquitinating GPX4, thereby inducing cell ferroptosis and apoptosis (11). Yang et al. discovered a significant correlation between TNBC subtype LAR tumors and ferroptosis through multi-omics sequencing, with the main ferroptosis inhibition pathway involving GSH, mediated by GPX4 (16). Additionally, studies have indicated that the combined levels of ACSL4/GPX4 in BC patients without a family history can serve as a novel predictor and prognostic biomarker for pathological complete remission in patients receiving neoadjuvant chemotherapy (116). Therefore, directly targeting GPX4 can also efficiently induce ferroptosis in TNBC cells.

### 4.3 Promoting Lip-ROS accumulation in TNBC

Ma et al. reported that siramesine and lapatinib can increase ROS production to induce ferroptosis in cells, while overexpression of CDO1 in breast cancer cells leads to a decrease in GSH levels, causing further accumulation of ROS, exacerbating ferroptosis in breast cancer cells (151). Additionally, studies have found that TNBC cells co-cultured with adipocytes exhibit resistance to ferroptosis, and this process depends on the fatty acid synthase ACSL3 secreted by adipocytes (88). Although the excessive accumulation of Lip-ROS plays a crucial role in ferroptosis research, it is still unclear whether the excessive Lip-ROS originates from the mitochondrial membrane, endoplasmic reticulum membrane, or other cellular membrane structures.

### 4.4 Increasing iron content in TNBC

Studies have shown that highly saturated Lf in MDA-MB-231 TNBC cell lines can increase cellular iron content and promote ROS generation, leading to cell ferroptosis. In contrast, low iron-saturation Lf can upregulate the expression of SLC7a11 and increase GSH production, thereby inhibiting the occurrence of cell ferroptosis (147). In addition, Yu et al. confirmed that the key gene after anti-HER2 treatment, Fibroblast Growth Factor Receptor 4 (FGFR4), can accelerate cysteine uptake and Fe^2+^ excretion through the β-catenin/TCF4 SLC7A11/FPN1 axis. The inhibition of FGFR4 can lead to an unstable iron pool accumulation, thus triggering cell ferroptosis (106). Therefore, increasing cellular iron content to induce ferroptosis may effectively prevent the malignant progression of BC patients. Regarding p53-mediated ferroptosis in TNBC cells, previous studies have shown that various tumor suppressor factors can promote cell sensitivity to ferroptosis. Thus, tumor suppression may be an inherent physiological function of ferroptosis (78). Research has found that p53 can induce not only cell apoptosis but also cell ferroptosis. Jiang et al. believed that p53 selectively inhibits the abnormal expression of the System Xc- subunit SLC7A11, significantly inhibiting GPX4 activity, thereby inducing cell ferroptosis (152). However, recent studies have found that the mutant p53 hot spot protein p53R175H can specifically bind to the transcription factor BACH1. This process can either relieve BACH1’s downregulation of SLC7A11, inhibit ferroptosis, promote tumor growth, or upregulate the expression of pro-migration targets and accelerate tumor metastasis (153). Therefore, personalized treatment plans are particularly important for different types of p53 mutations. In current BC ferroptosis research, p53 mainly promotes ferroptosis, but does this process also involve dual regulation? Further research may provide more precise personalized treatment plans for BC patients.

### 4.5 The role of ferroptosis in the treatment of TNBC

#### 4.5.1 Enhancing the effectiveness of chemotherapy

TNBC patients currently do not benefit significantly from traditional targeted therapies, making chemotherapy a viable option. However, tumor cells evading apoptosis led to chemotherapy resistance, posing a challenge in maximizing the therapeutic effects of drugs. Can inhibiting tumor cell proliferation through ferroptosis enhance the anticancer effects? Chen et al. found that the classic ferroptosis inducer Erastin, combined with tamoxifen, synergistically countered anticancer cells. Therefore, combining ferroptosis inducers and chemotherapeutic drugs may potentially address chemotherapy resistance in breast cancer cells (83). Ferroptosis also plays a crucial role in sensitizing radiotherapy. Holo-lactoferrin (Holo-Lf) is a saturated form of Lf. In combination therapies, Holo-Lf effectively increases the total iron content in TNBC cells, promoting the generation of reactive oxygen species (ROS) and the lipid metabolism byproduct malondialdehyde (MDA), leading to iron-mediated cell death (147). Furthermore, when Holo-Lf is combined with a 4 Gy radiation dose for treating MDA-MB-231 cells, there is a significant increase in lipid-ROS levels, suggesting that Holo-Lf enhances the sensitivity of tumor cells to radiation therapy (147).

#### 4.5.2 Enhancing the anti-cancer effects of natural compounds

With the inheritance and development of herbal and traditional Chinese medicine (TCM), especially the increasing role of Chinese herbal medicine in clinical treatment, the role of traditional Chinese medicinal materials in cancer treatment is becoming more significant. *Lycium barbarum* polysaccharides are extracted from the TCM *L. barbarum*, and studies have shown that Lycium barbarum polysaccharides had anticancer activities by indirectly mediating TNBC cell ferroptosis through the SLC7A11 and xCT/GPX4 pathways (154). This transforms into targeted modern Chinese medicine treatment for TNBC patients, providing new ideas for anticancer research. Shuganning injection (SGNI) is a patented TCM injection. It has been approved by the China Food and Drug Administration (CFDA) for use in the clinical treatment of liver diseases. It is also widely used in adjunct cancer therapy due to its liver protection and immune improvement properties. Research has shown that compared to non-TNBC cells and normal cells, SGNI can induce oxidative stress in TNBC cells in an HO-1-dependent manner and promote the accumulation of unstable intracellular iron pools, leading to TNBC cell ferroptosis (95). Furthermore, rutin (155), ginsenoside CK (156), ginsenoside Rh4 (157), and others have been confirmed to directly induce TNBC cell ferroptosis or interfere with different signaling pathways to inhibit tumor cell proliferation.

#### 4.5.3 Enhancing anticancer effects of novel drug delivery systems

In recent years, extracellular vesicles (EVs) have become a research hotspot both domestically and internationally, with studies showing that EVs play an essential role in inhibiting the malignant progression of tumor cells. Meanwhile, erastin, as an inducer of ferroptosis, has disadvantages such as poor water solubility and low bioavailability. Therefore, an effective drug delivery method is needed to overcome the obstacles of erastin. Yu et al. used erastin-loaded EVs (erastin@FA-exo) to efficiently deliver Erastin to the corresponding targets in MDA-MB-231 cells, thereby precisely and effectively inducing ferroptosis in MDA-MB-231 cells (158). Similarly, RSL3 has disadvantages such as low water solubility and poor biocompatibility, limiting the *in vivo* application of GPX4 inhibitors. To overcome traditional chemotherapy resistance in TNBC cells, researchers have developed an actively targeted small molecule self-assembled nano-drug. This drug can simultaneously deliver the chemotherapeutic drug camptothecin (CPT), iron catalyst (Fc), and GPX4 inhibitor (RSL3), forming a three-component nano-drug RSL3@CPT-SS-Fc. In the tumor environment with higher levels of GSH, the nano-drug can be broken down, causing cells to undergo both ferroptosis and apoptosis (159). Therefore, understanding the changes in ferroptosis caused by various pathways and TNBC tumor immune infiltration can further help us explore the diagnosis and treatment of TNBC from the perspective of ferroptosis. Further exploration of relevant pathways and functions can better elucidate the role of ferroptosis in TNBC, thus providing new insights and guidance for diagnosing and treating TNBC. Additionally, combining novel drug delivery systems to more accurately deliver drugs to their respective targets to enhance anticancer effects will become a novel therapeutic strategy in oncology.

## 5 Ferroptosis in HER2-positive breast cancer

HER-2 (Human Epidermal Growth Factor Receptor 2) is a critical oncogene that plays a significant role in the biological behavior of cancer. It is amplified and/or overexpressed in 14-30% of breast cancer cases, categorizing these as HER2-positive breast cancers, which are associated with poorer survival outcomes (160, 161). With the ongoing development of targeted therapies, the prognosis for HER2-positive breast cancer patients has improved to some extent. However, a portion of patients develop resistance to targeted drugs within a year, and the mechanisms underlying this resistance remain unclear (161). Recent studies have shown that ferroptosis is involved in the targeted treatment and resistance of HER2-positive breast cancer. For instance, Shi et al. discovered that ferroptosis biomarkers could predict tumor mutation burden (TMB), aiding in the prognosis of HER2-positive breast cancer patients. Combining these markers with TMB could allow for more accurate assessments of patient outcomes and provide new approaches for diagnosis and treatment (162).

In efforts to overcome HER2-positive breast cancer resistance through ferroptosis targeting, Zou et al. found that the upregulation of FGFR4 expression reduced ferroptosis in HER2-positive breast cancer, contributing to treatment resistance (106). Patient-derived xenografts and organoid models revealed that roblitinib, a selective FGFR4 inhibitor (also known as FGF-401), is effective in both intrinsic and acquired anti-HER2-resistant breast cancers (106). This study identified a mechanism of anti-HER2 resistance and proposed a strategy to overcome it by inhibiting FGFR4 in HER2-positive breast cancer.

Distant metastasis and recurrence remain the leading causes of breast cancer-related deaths (163), with brain metastasis being a specific risk factor in HER2-positive breast cancer. Due to resistance and the challenges posed by the blood-brain barrier, nearly half of the patients with brain metastases remain incurable. In a study using nude mice transplanted with HER2-high-expressing TBCP-1 cells, neratinib was found to induce ferroptosis in TBCP-1 cells, significantly inhibiting tumor growth and metastasis to the liver, lungs, and brain. These results indicate that neratinib may suppress distant metastasis in breast cancer through ferroptosis (91). Additionally, Zou et al. discovered that crVDAC3 reduces ferroptosis by preventing HSPB1 ubiquitination, contributing to trastuzumab deruxtecan resistance in HER2-low breast cancer (164).

## 6 Hormone receptor-positive breast cancer

A recent study discovered that in ER-positive and AR-positive breast cancer, MBOAT1 and MBOAT2 are regulated differently to inhibit ferroptosis, with MBOAT1 uniquely upregulated in ER-positive breast cancer. This upregulation is associated with ferroptosis resistance in ER-positive breast cancer (165). ChIP-qPCR analysis confirmed the binding of ER to the MBOAT1 ERE and further demonstrated that Fulvestrant (Ful) reduced this binding. Knockout of ESR1 or FOXA1 also decreased MBOAT1 expression (165). Therefore, MBOAT1 is a direct transcriptional target of ER. After ER degradation, endogenous MBOAT1 expression is significantly downregulated, rendering ER-positive breast cancer cells sensitive to ferroptosis (165). The study also found that Fulvestrant combined with IKE (a ferroptosis inducer) significantly inhibited tumor growth. In tumor tissues, Fulvestrant alone downregulated MBOAT1 expression, but only in combination with IKE did it upregulate PTGS2 expression. Thus, combining ER-targeting therapy with ferroptosis induction is a potential strategy for treating ER-positive breast cancer resistant to single ER-targeting therapies (165).

Tamoxifen resistance remains a major obstacle in the treatment of advanced breast cancer. In addition to its competitive inhibition of the ER signaling pathway, increasing ROS to impair mitochondrial function is critical to enhancing Tamoxifen efficacy. Xu et al. found that RelB-activated GPX4 inhibits ferroptosis and confers Tamoxifen resistance in breast cancer (166). In the field of epigenetics, it has been shown that targeting LINC00152 activates the cAMP/Ca(2+)/ferroptosis axis and overcomes Tamoxifen resistance in ER-positive breast cancer (167). Liu et al. discovered that ESR1 inhibits ionizing radiation-induced ferroptosis in ER-positive breast cancer cells through the NEDD4L/CD71 pathway (168). In terms of drug resistance, studies have shown that combining etoposide with erastin alters iron homeostasis by modulating IREB2/FPN1 expression in ER-positive breast cancer cells, inducing ferroptotic cell death (169).

## 7 Prospects

Ferroptosis is an interconnected network involving iron, selenium, amino acids, lipids, and redox chemistry, all of which play crucial roles in both physiological and pathological processes. As research into ferroptosis deepens, it has become evident that this network contributes significantly to the initiation, progression, invasion, and metastasis of breast cancer. This review summarizes the latest advances in understanding the molecular mechanisms related to ferroptosis in breast cancer, expanding our knowledge of the associated signaling and metabolic pathways involved in its pathology. Furthermore, with the growing identification of key molecular targets and regulatory mechanisms, such as gene transcription regulation and post-translational modifications involved in ferroptosis, targeting various aspects of this process may present novel therapeutic opportunities for breast cancer treatment. Current research suggests that ferroptosis exerts a dual effect on breast cancer, both promoting its development and serving as a potential therapeutic target to inhibit tumor progression.

Ferritinophagy, a key pathway for releasing free iron within cells, plays a crucial role in promoting ferroptosis by producing excess iron ions. However, further research is needed to fully elucidate the mechanisms of ferritinophagy in breast cancer. Additionally, many known ferroptosis-related targets have yet to be directly validated in breast cancer models. Future research should focus on investigating how ferroptosis affects various immune cells, such as neutrophils and macrophages from the innate immune system, and T cells and B cells from the adaptive immune system within the tumor microenvironment. This could offer insights into new therapeutic strategies and prevention methods for breast cancer.

Another key area of interest lies in identifying definitive biomarkers for ferroptosis. Although lipid peroxidation, iron accumulation, ROS, GPX4 expression, and cell viability have been proposed as indicators of ferroptosis, no specific biomarkers have been confirmed to date. While other forms of programmed cell death have well-defined markers, finding distinct biomarkers for ferroptosis would greatly enhance our understanding of its biological role. Despite significant advances in understanding ferroptosis regulation, the exact mechanisms by which cells undergo death remain unclear. The furthest downstream step identified so far involves the uncontrolled peroxidation of polyunsaturated fatty acid-containing phospholipids (PUFA-PLs), which may lead to membrane damage or rupture, compromising membrane integrity. Interestingly, recent studies suggest phospholipids with two PUFA tails are more effective in driving ferroptosis, hinting that lipid crosslinking might contribute to membrane damage. Clarifying the precise mechanisms through which ferroptosis leads to cell death will be a major focus in the coming years.

Furthermore, the interplay between inflammation and ferroptosis within the breast cancer microenvironment and how these processes contribute to cancer development requires further investigation. The underlying mechanisms still need to be elucidated in future research. When using small molecule compounds in animal models, it is critical first to establish the appropriate formulation and delivery methods to achieve meaningful pharmacokinetic and pharmacodynamic responses in target tissues. Many small molecule probes that perform well *in vitro* are unsuitable for *in vivo* studies due to poor solubility, stability, and/or pharmacokinetics. Standard methods to improve these factors often prove inadequate; some should be avoided. For instance, RSL3 has poor solubility, making it difficult to measure its pharmacokinetics in mice, which limits its utility for direct injection into tissues or tumors. Similarly, erastin suffers from low solubility and metabolic stability, and ferrostatin-1 is unsuitable for *in vivo* research. However, liproxstatin-1 and vitamin E can be used as ferroptosis inhibitors in animal studies. The application of iron chelators to inhibit ferroptosis *in vivo* or should be interpreted cautiously, as iron depletion may have unintended consequences beyond ferroptosis inhibition. It remains unclear whether some observed effects are due to iron chelation or the compounds’ off-target effects. Therefore, future studies should carefully evaluate these results, especially with an eye toward clinical translation.

In the context of breast cancer’s immune microenvironment, iron metabolism and lipid peroxidation may induce ferroptosis, but the precise mechanisms remain to be clarified. It is important to investigate how both classical pathways, such as GPX4/ACSL4 ferroptosis signaling, and non-classical pathways, like GPX4-independent ferroptosis, influence breast cancer progression, invasion, and metastasis. Future research should focus on combining ferroptosis-targeting strategies with other therapies to overcome tumor resistance and enhance antitumor immunity. For example, recent studies suggest that the luminal androgen receptor subtype is highly sensitive to ferroptosis, and combining GPX4 inhibitors with PD-1 inhibitors may offer new therapeutic options. These findings underscore the potential of targeting ferroptosis as a promising strategy for breast cancer treatment, presenting both therapeutic potential and research value in future clinical applications.
